# Exploring Sleep Behaviors and Routines, and Mindfulness Practices Among Seniors Residing in Low-Income Housing

**DOI:** 10.1093/geroni/igaf122.3096

**Published:** 2025-12-31

**Authors:** Chidera Ejikeme, Robert Linscott, Stuart Quan, Matthew Weaver, Charles Czeisler, Rebecca Robbins

**Affiliations:** Brigham and Women’s Hospital, Boston, Massachusetts, United States; City of Boston, Boston, Massachusetts, United States; Harvard Medical School, Boston, Massachusetts, United States; Harvard Medical School, Boston, Massachusetts, United States; Brigham and Women’s Hospital, Boston, Massachusetts, United States; Brigham and Women’s Hospital, Boston, Massachusetts, United States

## Abstract

Poor sleep is common among older adults and the general population. Low socioeconomic status is associated with sleep disturbance and short sleep duration. Sleep disturbance is associated with an increased risk for Alzheimer’s disease and incident dementia. Evidence indicates that mindfulness can improve sleep duration and quality. We conducted a qualitative study of sleep and mindfulness practices among seniors in a low-income housing facility in the US Northeast. Participants completed the Montreal Cognitive Assessment (MoCA), a brief demographic questionnaire, and an interview about sleep and mindfulness. Interviews were audio-recorded and transcribed verbatim. Qualitative analysis proceeded in accordance with the Constant Comparative Method. Among participants (n = 12), average age was 70.1 (s.d.=6.7); 83.3% of participants were White and 16.7% were Black. Participants were 50% male. Half the sample reported sleeping 7 hours, 25.0% 6 hours, and 25% 8 or more hours. Average score on the MoCA was 23.3 (s.d.=4.03). Common issues related to healthy sleep included taking long naps, nighttime television use, waking from sleep and having trouble falling back asleep, and nighttime checks interrupting sleep conducted by staff members of the residents. Participants reported limited awareness of mindfulness, but in one case, that learning mindfulness skills was ‘life changing.’ Several reported attempting mindfulness, but being unable to implement a regular practice. Another resident mentioned interest in social support for mindfulness practice. Results from this study highlight sleep struggles, and opportunities for tailoring sleep and mindfulness content in programs for older adults residing in low-income housing.

